# Communicating Intelligence Research

**DOI:** 10.3390/jintelligence8040040

**Published:** 2020-11-19

**Authors:** Jonathan Wai

**Affiliations:** Department of Education Reform and Department of Psychology, University of Arkansas, Fayetteville, AR 72701, USA; jwai@uark.edu

**Keywords:** science communication, teaching, education, open inquiry, research practice gap

## Abstract

Despite intelligence research being among the most replicable bodies of empirical findings—a Rosetta stone across the social sciences—the communication of intelligence research with non-intelligence researchers and the public remains a challenge, especially given ongoing public controversies throughout the history of the field. Hunt argued that “we have a communication problem.” This article is a call for intelligence researchers to consider communication at multiple levels—communication with other intelligence researchers, communication with non-intelligence researchers, and communication with the public, defined here as policymakers, practitioners, students, and general readers. It discusses ongoing tensions between academic freedom and social responsibility and provides suggestions for thinking about communication and effective research translation and implementation of intelligence research from the frameworks of science and policy research communication. It concludes with some recommendations for effective communication and stresses the importance of incentivizing more scholars to responsibly seek to educate and engage with multiple publics about the science of intelligence.

“We have a communication problem” (Hunt 2009, p. 324).

## 1. Introduction of the Problem

The research on intelligence, from a purely scientific perspective, is among the most robust literatures in all of the social sciences (Arvey et al. 1994; Carroll 1997; Deary 2020; Jensen 1998; Neisser et al. 1996). At least within the community of researchers around the world who openly acknowledge this enormous body of evidence accumulated to date, then, whether intelligence or cognitive abilities are measurable and have real world consequences is not, at least in my view, the most crucial debate. Of course, there remain healthy disagreements in the field about various aspects of intelligence, and we still have much to learn about intelligence and how it might be most fruitfully applied. Yet, intelligence researchers often find themselves facing many people outside of the field (including the general public) holding strong misconceptions or even distorting the facts about intelligence. Thus, this article seeks to build from Hunt’s (2009) point that the field has a communication problem as a way of expanding the list of challenges or problems that intelligence researchers face in conducting scientific inquiry and stressing that communication has been a neglected topic for the field.

Because intelligence research is multidisciplinary and the construct—especially when conceptualized and measured as general intelligence or *g*—can be considered a Rosetta stone across the social sciences (Jensen 1998, 2006), it (should) have influence in numerous other fields. Some fields, such as industrial/organizational (I/O) psychology (e.g., Schmidt and Hunter 2004; Kuncel et al. 2004), are one step removed from intelligence research but have incorporated it into their discipline and training (they just call it general mental ability, or GMA). Other fields, such as gifted education, are somewhat accepting of intelligence as a core aspect, but still it remains a minority perspective (e.g., Thompson and Oehlert 2010; Wai and Worrell 2015; Warne 2015). On the flipside, there are other fields, such as epidemiology (e.g., Gottfredson 2004) and education (e.g., Wai et al. 2018) in which there has simply been little to no integration of intelligence research at all, and even strong resistance or just open absence of acknowledgment throughout history (e.g., Maranto and Wai 2020). In the field of psychology, beyond the I/O subdiscipline, it does not appear that intelligence research is really fully accepted as indicated by the number of jobs in traditional psychology, education, or other departments who employ intelligence researchers. Moving outside psychology to the broader social sciences or other areas of science does not necessarily show intelligence research being integrated strongly in a systematic way. One indicator intelligence research is not well accepted by mainstream psychology is the relatively low number of faculty positions, at least in the U.S., recruiting for intelligence as a specialty (e.g., in the U.S. the Psychology Job Wiki (psychjobsearch.wikidot.com), throughout recent years in particular, almost uniformly does not have any positions explicitly asking for intelligence as a specialty). Additionally, though not typically seen this way by intelligence researchers, the lack of accuracy of how intelligence research is represented in general psychology textbooks (Warne et al. 2018) is another indicator of mainstream acceptance within the field of psychology broadly. However, more systematic research on the representation of intelligence as mainstream in psychology has also not yet been conducted and would be useful information.

The history of intelligence research has been simultaneously filled with enormous empirical advancement alongside a number of resurfacing public controversies (see Jensen 1969; Gottfredson 2010a; Herrnstein and Murray 1994; see Carl and Woodley 2019; Rindermann et al. 2020 for recent reviews). Some intelligence researchers have argued that given how intelligence research has the potential to be misused due to its complicated and sometimes unfortunate history, that it is crucial to be careful about how such research is conducted and disseminated (e.g., Martschenko et al. 2019). This type of concern about the history of intelligence research may have even prompted the *Journal of* Intelligence (2020) editors to openly state on their “aims” page that certain types of research questions, as determined at the discretion of the editors, will not be accepted: “The journal will not consider manuscripts that present results or conclusions with mixed language, with misleading wording or with insufficient supporting data that may therefore lead to or enhance political controversies; and the editors will judge whether that is the case.” Haier’s (2020) editorial stance in *Intelligence* states that “Our responsibility is to publish the best quality studies we can to elucidate all aspects of human intelligence research. In our view, publishing empirical data, along with clear explanations of what the data mean and what they do not mean, is the only basis for reasoned discussions about what intelligence is and why it is important.”

Thus, there is debate within the field of intelligence itself on what are acceptable topics to conduct research on and what are not and what should even be communicated. Some intelligence researchers believe that all questions should be openly pursued and communicated (e.g., Carl and Woodley 2019; Jensen 1998). Other researchers have noted that certain questions in intelligence research are a bit like playing with fire (e.g., Hunt and Lubinski 2005; Martschenko et al. 2019; Sternberg 2005). It is clear that intelligence research often comes under attack for reasons that have nothing to do with the integrity of the science, but more to do with the possible social implications and misuse when it comes to policy (Martschenko et al. 2019). 

The National Academies of Sciences, Engineering, and Medicine (2017, p. 53), includes a very pertinent section on “The nature of science-related public controversies,” and notes that though these public controversies are diverse, there are three main features they typically share: 1. “Conflicts over the beliefs, values, and interests of individuals and organizations, rather than simply a need for scientific knowledge, are central to the debate”; 2. “The public perceives uncertainty either in the science or in its implications or as a result of communicators making different and sometimes contradictory statements in the public sphere”; 3. “The voices of organized interests and influential individuals are amplified in public discourse, making it difficult for the state of scientific evidence to become clearly known.” Certain aspects of intelligence research being publicly controversial becomes much clearer when viewed through this lens.

This article builds on the perspective of Hunt (2009, p. 324), who emphasized that in the intelligence research community “we have a communication problem.” The article first provides a framework for thinking about communication of intelligence research in terms of scholarly aspects (e.g., Cohen 2019) but also more broadly (Lewis and Wai 2020; Tseng 2012). This includes communicating research findings with other intelligence researchers, then moves to discussing the communication of intelligence research with other subdisciplines of social science and beyond, and finally discusses the communication of intelligence research with the public, which is defined here as policymakers, practitioners, students, or general readers. The perspective of this piece is primarily from that of an intelligence researcher working in the U.S. One aim of this article is to try to provide a framework for intelligence researchers themselves to understand the multiple channels of communication that exist and where intelligence research runs into conflict and controversy (for detailed explanation of specific topics that have long been known to be contentious, see (Carl and Woodley 2019; Martschenko et al. 2019; Rindermann et al. 2020). Another aim is to start a field-wide conversation on this topic, given what I see as its importance to long-term sustainability of the continually evolving core group of researchers studying intelligence in the years to come.

History tells us that because of its sensitive social implications, intelligence research will continue to be publicly controversial even if it is scientifically uncontroversial (National Academies of Sciences, Engineering, and Medicine 2017). I will try to encourage intelligence researchers to think about strategies for ensuring the survival of the discipline, by thinking about what we are communicating and how we are communicating with others. At the heart of this consideration is responsible engagement on what gets communicated (Lewis and Wai 2020; Tseng 2012) while simultaneously balancing the historical and enduring need for academic freedom and open inquiry (Haier 2020; University of Chicago 1967; Zimmer 2015). Communication occurs at multiple levels and is not just about intelligence researchers getting others to understand what the current facts or weight of evidence of our field have to offer through getting the message right. It is as much or even more about seeking to ensure those we seek to communicate to or with are even willing to listen (in some cases they are not). Communication requires all parties involved to actively want to do so. It requires a genuine exchange, acceptance, and integration of information (Conaway 2019). As Tseng (2012, p. 12) explains regarding the uses of research in policy and practice, “we do not simply face a communications problem of better conveying research; nor is it merely a dissemination problem of better distributing research. Translation is critical, and we should reflect more intentionally on who makes for the best translators and how to create productive contexts for translation.”

I hope that by initiating a conversation within the field we can more effectively and openly pursue questions related to intelligence research and also more deeply understand and consider the context and consequences of the research we conduct from the lens of society at any place and at any given time in history.

## 2. Communicating with Other Intelligence Researchers

It is important to communicate with other researchers who are a part of the International Society of Intelligence Research (ISIR) and publish in *Journal of Intelligence*, *Intelligence* and additional journals that publish individual differences research, and can be considered the core community of intelligence researchers. However, there are other researchers who also study intelligence similarly defined but have other home sub-disciplines (e.g., personality, behavioral genetics, industrial/organizational, expertise, gifted education). These researchers have a diverse array of views about intelligence research, but on the whole there is a rough general consensus on a wide range of topics (Rindermann et al. 2020). There is healthy communication of disagreement between intelligence researchers because we have a shared goal of uncovering the truth about all aspects of intelligence and its implications for society. Thus, though there remain disagreements about what should be studied and/or communicated within the field itself, largely the communication of scientific information with each other is not the most salient issue.

## 3. Communicating with Non-Intelligence Researchers

Gottfredson (2010b) explained what it was like to explore across and learn from different disciplines (p. 28): “Disciplines literally speak different languages, where the same word can mean different things—usually that discipline’s favorite part of the metaphorical elephant…” All this makes for confusing and fraught cross-disciplinary communication. Doing interdisciplinary research is like moving to a foreign land with a different history, language, and culture. It takes time, exposure, and effort—immersion—to finally “catch on.” Because the field of intelligence has its own methodological history and approach as well as accumulated scientific understanding and shared norms that may be different from that of other fields, in addition to having different terms and language, being able to communicate the findings of intelligence research requires an academic from another discipline to be open to thinking about the body of research from intelligence in the context of their own discipline, methods, history, and approach. To a large degree, learning the language of a new discipline often requires immersion in that discipline (Institute of Medicine 2005; Lyall 2019) Gottfredson (2010b) explains how she has been able to move into other fields and learn their language to be able to integrate it with the language of the field of intelligence; however, being able to communicate the importance of intelligence research with non-intelligence researchers largely would require them to do what Gottfredson did (who was initially trained as a sociologist), coming into our world to learn from us. Another example is Flynn (1987, 2007) who was trained in political science and helped the intelligence community to more deeply investigate what has come to be known as the Flynn effect or rising IQ scores across the decades. Yet another example is (Jones 2016; Jones and Schneider 2006) who drew from the field of intelligence and brought it into economics. Beyond these examples, I am not sure how often scholars outside our field seek to learn about our mainstream findings (and perhaps a systematic analysis of the researchers from other fields who draw from intelligence research would be worthwhile to conduct). However, given the nature of academia and its reward system, it is much harder to move across disciplines than it is to stay within one’s discipline.

## 4. Communicating with the Public: Policymakers, Practitioners, Students, and General Readers

Scholars within the field of intelligence convened a Special Issue in *Intelligence* to discuss the teaching of intelligence and in particular wondered why more classes on intelligence were not taught (Detterman 2014). At least within the U.S, but also probably outside of it, as Hunt (2009, 2010) has pointed out, some if not most of the findings from the field of intelligence are not socially palatable. Chabris (1998) published an influential article in *Commentary* magazine summarizing the research on intelligence and its importance in response to the controversy surrounding the publication of *The Bell Curve* (Herrnstein and Murray 1994), and expressed the concern that, though in terms of the scientific debate on intelligence, researchers were on solid ground, outside of the scientific world where evidence matters much less when it comes to battles, such as in the media, the field of intelligence was losing the war.

The public can broadly be considered an amalgamation of different publics, people like policymakers, practitioners, current and potential students, and general readers (Conaway 2019; Tseng 2012). These are people who largely have no scientific training in any specific discipline and thus know very little about the scientific body of evidence that has accumulated in any field, including the field of intelligence research. The purpose of Arvey et al. (1994), Neisser et al. (1996), Carroll (1997), and Chabris (1998) were all to seek to educate other researchers but especially the public about misconceptions in the media about intelligence research. Chabris (1998) points out that engagement and education of the public is important for the purpose of ensuring the public is aware of the appropriate scientific information to use in decision making, and to correct the scientific record in their eyes (Chabris 2013; also see Pinker 2009).

In response to Chabris (1998), Deary et al. (1998) noted:

“For those of us who are researchers in the field, however, Mr. Chabris offers a doleful prediction: though we win all the intellectual battles, we shall lose the war. Why? Because, he suggests, up and coming researchers are dissuaded from studying intelligence differences by the bad press the field has received from a series of high-profile attacks, including Stephen Jay Gould’s *The Mismeasure of Man* and the responses to Richard J. Herrnstein and Charles Murray’s *The Bell Curve*. But we think the propaganda war on intelligence differences need not be won; in fact, one should not even engage in the contest, for two reasons. First, it takes place outside the realm of scientific discourse; its weapon is rhetoric and its shield is public ignorance. Winning such a war would not equate with being correct, which is all that should matter in science. Second, within the relevant scientific community, the debate over the importance of traditional-style research into human intelligence differences is nonexistent.”

Deary essentially expresses that which is true: whatever the public (or even other research domains) thinks about the field of intelligence does nothing to change whether its scientific base is correct in the eyes of those conducting mainstream research. However, the bad press, good press, neutral press, or lack of press our field receives, depending upon how high profile it is or how widespread and consistent, most certainly has consequences not only for researchers themselves who currently study intelligence in universities, but also potential future students whose main exposure to the topic, like the public, largely comes from the media or popular writers. For example, given the current hiring freezes of new faculty, the already limited faculty jobs for intelligence researchers will likely become quite restricted for years to come (e.g., Woolston 2020). This matters for the recruitment and retention of new students into the field, since smart students are driven not only by their interests, but also the job opportunities for certain domains both within and outside of academia after graduation (e.g., Brennan 2020).

Deary (2020) has since sought to educate through clear explanation of intelligence research and its importance and there is no question Deary is an outstanding scientist. However, the point made by Chabris (1998, 2013) that we should engage in responsibly educating the public about intelligence research remains correct to this day, at least in my view. This is clearly corroborated from the outside (non-scientist) perspective of a distinguished science writer speaking about communicating science to non-specialist audiences (Siri Carpenter, editor of *The Open Notebook:* https://www.theopennotebook.com/): “Science writers and scientists share some core goals in common. The aim of any storytelling or writing, no matter who you are, is to draw people into your story, to provide them with clear and accurate information, to present it in a way that engages people to keep reading, and to leave them with some kind of trace of the whole experience. That’s true whether your purpose is to spark some kind of action, or whether it’s simply to inform or entertain. There’s no point to any of it if no one is going to read it or understand it or remember it. And that’s true whether you are a professional science writer or a scientist who wants to communicate with your peers and students, or with those who review your grant proposals, or with the general public” (Carpenter and Wai 2020). As noted earlier, communicating our findings or determining a consensus around multiple topics within the field of intelligence has not really been the most salient issue (e.g., Neisser et al. 1996). The issue has largely been about effectively communicating with those outside our field so that they might even be open to current empirical findings based on the weight of the evidence about our discipline, especially when these findings are publicly contentious (Hunt 2009; National Academies of Sciences, Engineering, and Medicine 2017; Tseng 2012). Tseng (2012) goes further to argue that the challenges for research to impact policy and the public go well beyond communication and dissemination but are also about who is doing the translation and how productive contexts for translation can be developed. For example, in education policy, a research-practice partnership (Conaway 2019) is a form of long-term agreement where researchers work alongside practitioners who directly work with schools to initiate research questions that will have a higher likelihood of being useful both in terms of solving problems of practice and also making scientific advances.

## 5. What Are Some Characteristics of Effective Ambassadors for Communicating Intelligence Research?

*Some examples of effective public communication from intelligence researchers themselves and why I think that is so. This list is by no means exhaustive*.

Stuart Ritchie’s book *Intelligence: All That Matters* (Ritchie 2016) is a very effective short introduction written in plain language and in an engaging, lighthearted, and humorous style that tackles intelligence and why it matters in a way that works. Ritchie has also been very effective at discussing intelligence in other public venues (on *Vox*: (Resnick 2016; *The Psychology Podcast*) (Kaufman 2020). Richard Haier’s book *The Neuroscience of Intelligence* (Haier 2017) is also a very effective review of intelligence research but more focused on neuroscience aspects. Haier is very good at using plain language and being very clear but does so sensitively. This interview (Haier and Kaufman 2020) is an excellent example of how Haier directly addresses contentious issues but does so in a way that is fair and balanced. Scott Barry Kaufman is the author of numerous books, some of which tackle the topic of intelligence, such as *Ungifted: Intelligence Redefined* (Kaufman 2013). Kaufman is also a regular contributor to *Scientific American* and often will have guests on *The Psychology Podcast* like Ritchie, Haier, and others, to discuss various aspects about intelligence, has reached millions of people worldwide, and is worth learning from. Paige Harden has been successful at discussing the intersection of intelligence and genetics research (*Sam Harris Podcast* (Harden and Harris 2020; *NBC News:* ) (Yuhas 2020). Kathryn Asbury has also been effective at sensitively communicating how intelligence research and genetics can be important for education (*G is for Genes* (Asbury and Plomin 2013; *Times Educational Supplement*) (Asbury and Severs 2018). Andrew Conway writes “channel g” for *Psychology Today* (Conway 2020) and has tackled the University of California college admissions topic rigorously but with an appropriate and fair tone. Christopher Chabris is an excellent example of someone who writes publicly for very prominent U.S. publications about controversial topics such as intelligence (Chabris 1998), but does so in a way that is focused on the evidence and facts and seeks to be engaging, and is a good model to follow (*Slate* (Hambrick and Chabris 2014; *Los Angeles Times*) (Chabris and Wai 2014; *The Washington Post*) (Wai et al. 2019). I also have been writing for the past decade, and some core articles for the public can be found at: jonathanwai.net/writing. My style is not the same as the style of all these examples of effective communicators provided here, but in general these are, at least in my opinion, a sampling of useful models to learn from and follow for others interested in engaging in a way that can lead to educating and communicating with others about intelligence thoughtfully and sensitively.

*Some suggested guidelines for communicating with various audiences about intelligence research. What makes a good ambassador for communicating intelligence research*?

*Someone who recognizes they are a representative not only of themselves and their own research but ultimately the global intelligence research community*. First and foremost, it is important that, when one chooses to engage, they recognize that how they are perceived is a reflection not only upon themselves but also the broader scientific community that seeks to study intelligence research. To the extent that any individual researcher stirs controversy—deliberately or otherwise—this can have negative consequences for all intelligence researchers, whether for acceptance of intelligence research in psychology or other fields, for jobs for the next generation of scholars, or for the willingness of the public to learn about all aspects of intelligence research, some of which does not touch upon sensitive issues and in fact could be fruitfully used to help people. This also means, as Haier (2020) noted in a recent editorial for the journal Intelligence that there are some people who may not be good ambassadors for intelligence research because they are not careful in the way they represent the community. This means that we should encourage many more voices from the intelligence community to seek to responsibly engage so that no handful of voices become the only representatives of intelligence research to the public.

*Someone whose main goal is to educate about (their own interpretation of) the current research base on intelligence.* A good ambassador is one who first recognizes that the public likely does not know about the current evidence base surrounding intelligence (just as the public likely does not know about the current evidence base about many specialized fields of knowledge). Second, this ambassador understands that, at least in the U.S., most people have often been misinformed about intelligence research. Thus a good goal for an ambassador of intelligence research is to seek to educate, not only about one’s own research, but the broader body of research on intelligence, and what the current weight of the evidence is, and to honestly acknowledge that this evidence base can and does shift as we learn new things.

*Someone who understands that communicating scientific findings effectively requires an appreciation of the broader layers of context (e.g., political, scientific, academic, pragmatic) within which findings are considered*. This article was set up purposefully to discuss communication at different levels to highlight that audiences and publics differ and that communication with those audiences may have different challenges depending upon various contexts, including political ones. For example, for the field of education policy, of which I now am I part of and seek to introduce intelligence research into, politics has historically always been and will continue to be the main driving force within which even education scientists have to operate within (Greene and McShane 2018), and many policy decisions are not evidence driven (Whitehurst 2019). Additionally, within education policy is the issue that even when scientific findings are rigorous and have the potential to scale and be used to help children in theory, in many cases implementation of those findings evaporates once they are introduced to schools (e.g., Maranto and Wai 2020; Tyack and Cuban 1995). Most scientists tend to communicate largely with other scientists and often forget that this is a community that speaks a very specialized and specific language, and that once one leaves the domain of expertise many outside that domain are unlikely to understand (and should in many cases not be expected to understand). One strategy that may be helpful for intelligence researchers is to start more effectively explaining to the public why what they do can be useful (Epstein 2016). This is why communication matters and should be taken much more seriously by any academic community that seeks to help their science matter. This is also why I believe that public communication by scientists themselves should be incentivized and rewarded and that we should seek to train and encourage future generations of intelligence researchers to publicly engage (Baron 2010; Chabris 1998; Wai and Miller 2015), primarily because it is necessary for the future of the field itself to communicate what is replicable and correct about our field in way that is in full consideration of the wider context (e.g., Lewis and Wai 2020).

## 6. Communicating with the Future of Intelligence Research in Mind

At a time when psychology and the broader social sciences are struggling with a replication crisis (Open Science Collaboration 2015), the research on intelligence and its implications for society is among the most replicable domains across the social and behavioral sciences. Thus, the fact that intelligence as a field remains under fire at present for identical issues as those that continue to resurface across the decades suggests that Hunt (2009) is correct, we very likely have a communication problem. The goal of this short piece has been to provide a framework to think about communication of intelligence research at multiple levels, and how that might shed some light on the historical issue of intelligence not being well integrated into other academic disciplines and also not well understood or accepted by the general public. As a field, it is true that our first priority should be to ensure that we seek to build our scientific understanding of intelligence, but this does not preclude the need to ensure the institutional and public support for new researchers to be able to pursue intelligence research for their careers. For example, we need mainstream psychology, education, or other departments to hire and support the next generation of intelligence researchers, which comes from adjacent more mainstream fields (e.g., social/personality, developmental, health, educational, quantitative, I/O, cognitive) accepting the current empirical findings from intelligence and not being turned off by the public and academic controversies. In fact, responsible scientific engagement may be more critical than ever for the field of intelligence research as it seeks to maintain its reputation in academia and among the broader public (Lewis and Wai 2020), and this can even be viewed as an important way of teaching about intelligence (Detterman 2014)—students, after all, are simply members of the public who happen to be in college. One might enquire after the best way to communicate intelligence research, given the challenges of reaching other non-intelligence researchers and the public. The broad theme of responsible science engagement (Lewis and Wai 2020) is probably the most valuable approach, considering that research within the field, especially when that research can have societal consequences and implications, needs to be communicated with a sensitivity and genuine acknowledgment of social responsibility. The Appendix of Lewis and Wai (2020) provides some resources on getting started communicating about psychology broadly, and the earlier section providing some examples of effective ambassadors for the field may be helpful. The National Academies of Sciences, Engineering, and Medicine (2017) has a section devoted to “understanding the special complexities of communicating science in the face of public controversy,” which lays out multiple questions surrounding how much research has not yet been conducted in this area, with a focus on understanding the often idiosyncratic origins and dynamics of fields of research becoming controversial. The authors of this important report note that such research is important for improving communication of any given field.

Of course, concerns about social responsibility and sensitive communication should be balanced by the need for academic freedom and open inquiry (Haier 2020; University of Chicago 1967; Zimmer 2015) so that all intellectual perspectives are encouraged (Duarte et al. 2015). Estes (1992), then the editor of *Psychological Science*, noted that “Somehow a balance must be found between the need for free exchange of research results among scientists concerned with intelligence and the need to be sure that no segment of our society has reason to feel threatened by the research or its publication.” For the long-term survival of the field, these are the kinds of considerations and conversations about intelligence research we do need to have. Whether we like it or not, human biases, irrational responses to empirical findings, and institutional and social forces influence how and whether we are able to conduct research into intelligence and whether that is sustainable into the future (National Academies of Sciences, Engineering, and Medicine 2017). As the sociologist of science Merton (1973, p. 267) pointed out, “An institution under attack must re-examine its foundations, restate its objectives, seek out its rationale. Crisis invites self-appraisal.” As a community, perhaps it is time to consider the topic of communication for the sustained future of the field of intelligence.

## References

[B1-jintelligence-08-00040] Arvey Richard D., Bouchard Thomas J., Carroll John B., Cattell Raymond B., Cohen David B., Dawis Rene V., Willerman L. (1994). Mainstream science on intelligence. The Wall Street Journal.

[B2-jintelligence-08-00040] Asbury Kathryn, Plomin Robert (2013). G Is for Genes: The Impact of Genetics on Education and Achievement.

[B3-jintelligence-08-00040] Asbury Kathryn, Severs J. (2018). Genetics with Dr. Kathryn Asbury. Times Educational Supplement Podagogy Podcast.

[B4-jintelligence-08-00040] Baron Nancy (2010). Escape from the Ivory Tower: A Guide to Making Your Science Matter.

[B5-jintelligence-08-00040] Brennan Jason (2020). Good Work If You Can Get It: How to Succeed in Academia.

[B6-jintelligence-08-00040] Carl Noah, Woodley Michael A. (2019). A scientometric analysis of controversies in the field of intelligence research. Intelligence.

[B7-jintelligence-08-00040] Carpenter Siri, Wai Jonathan (2020). What Scientists Can Learn from Science Writers. Psychology Today.

[B8-jintelligence-08-00040] Carroll John B. (1997). Psychometrics, intelligence, and public perception. Intelligence.

[B9-jintelligence-08-00040] Chabris Christopher F. (1998). IQ since “The Bell Curve.” *Commentary* 106: 33–40. https://www.commentarymagazine.com/articles/christopher-chabris/iq-since-the-bell-curve/.

[B10-jintelligence-08-00040] Chabris Christopher F. (2013). Book review: ‘David and Goliath’ by Malcom Gladwell. The Wall Street Journal.

[B11-jintelligence-08-00040] Chabris Christopher F., Wai Jonathan (2014). Hire Like Google? For Most Companies, That’s a Bad Idea. Los Angeles Times.

[B12-jintelligence-08-00040] Cohen Philip N. (2019). Scholarly Communication in Sociology. Open Sociology.

[B13-jintelligence-08-00040] Conaway Carrie (2019). Maximizing research use in the world we actually live in: Relationships, organizations, and interpretation. Education Finance and Policy.

[B14-jintelligence-08-00040] Conway Andrew R. A. (2020). Channel *g*: Current Findings and Theories about Intelligence. Psychology Today.

[B15-jintelligence-08-00040] Deary Ian J. (2020). Intelligence: A Very Short Introduction.

[B16-jintelligence-08-00040] Deary Ian J., Caryl Peter G., Austin Elizabeth J. (1998). Does IQ matter?. Commentary.

[B17-jintelligence-08-00040] Detterman Douglas K. (2014). Special section: You should be teaching intelligence. Intelligence.

[B18-jintelligence-08-00040] Duarte José L., Crawford Jarret T., Stern Charlotta, Haidt Jonathan, Jussim Lee, Tetlock Philip E. (2015). Political diversity will improve social psychological science. Behavioral and Brain Sciences.

[B19-jintelligence-08-00040] Epstein David (2016). Reporting on Individual Differences: When Intuition Reigns.

[B20-jintelligence-08-00040] Estes William K. (1992). Postscript on ability tests, testing, and public policy. Psychological Science.

[B21-jintelligence-08-00040] Flynn James R. (1987). Massive IQ gains in 14 nations: What IQ tests really measure. Psychological Bulletin.

[B22-jintelligence-08-00040] Flynn James R. (2007). What Is Intelligence? Beyond the Flynn Effect.

[B23-jintelligence-08-00040] Gottfredson Linda S. (2004). Intelligence: Is it the epidemiologists’ elusive “fundamental cause” of social class inequalities in health?. Journal of Personality and Social Psychology.

[B24-jintelligence-08-00040] Gottfredson Linda S. (2010a). Lessons in academic freedom as lived experience. Personality and Individual Differences.

[B25-jintelligence-08-00040] Gottfredson Linda S. (2010b). Pursuing patterns, puzzles, and paradoxes. General Psychologist.

[B26-jintelligence-08-00040] Greene Jay P., McShane Michael Q. (2018). Failure up Close: What Happens, Why It Happens, and What We Can Learn from It.

[B27-jintelligence-08-00040] Haier Richard J. (2017). The Neuroscience of Intelligence.

[B28-jintelligence-08-00040] Haier Richard J. (2020). Academic freedom and social responsibility: Finding a balance. Intelligence.

[B29-jintelligence-08-00040] Haier Richard J., Kaufman Scott Barry (2020). Richard Haier on the Nature of Human Intelligence. The Psychology Podcast.

[B30-jintelligence-08-00040] Hambrick David Z., Chabris Christopher (2014). Yes, IQ Really Matters. Slate.

[B31-jintelligence-08-00040] Harden Kathryn Paige, Harris Sam (2020). A conversation with Kathryn Paige Harden. https://samharris.org/podcasts/212-july-29-2020/.

[B32-jintelligence-08-00040] Herrnstein Richard J., Murray Charles (1994). The Bell Curve: Intelligence and Class Structure in American Life.

[B33-jintelligence-08-00040] Hunt Earl (2009). Good news, bad news, and a fallacy: A review of Outliers: The story of success. Intelligence.

[B34-jintelligence-08-00040] Hunt Earl (2010). Human Intelligence.

[B35-jintelligence-08-00040] Hunt Earl, Lubinski David (2005). 2005 International Society for Intelligence Research (ISIR) Distinguished Contribution Interview with Earl Hunt. https://www.youtube.com/watch?v=W2kZAkbbIOw.

[B36-jintelligence-08-00040] Institute of Medicine (2005). Facilitating Interdisciplinary Research.

[B37-jintelligence-08-00040] Jensen Arthur (1969). How much can we boost IQ and scholastic achievement?. Harvard Educational Review.

[B38-jintelligence-08-00040] Jensen Arthur (1998). The g Factor: The Science of Mental Ability.

[B39-jintelligence-08-00040] Jensen Arthur (2006). Clocking the Mind: Mental Chronometry and Individual Differences.

[B40-jintelligence-08-00040] Jones Garett (2016). Hive Mind: How Your Nation’s IQ Matters so Much More Than Your Own.

[B41-jintelligence-08-00040] Jones Garett, Schneider W. Joel (2006). Intelligence, human capital, and economic growth: A Bayesian averaging of classical estimates (BACE) approach. Journal of Economic Growth.

[B42-jintelligence-08-00040] Journal of Intelligence (2020). Aims. https://www.mdpi.com/journal/jintelligence/about.

[B43-jintelligence-08-00040] Kaufman Scott (2013). Ungifted: Intelligence Redefined.

[B44-jintelligence-08-00040] Kaufman Scott (2020). The Psychology Podcast. https://scottbarrykaufman.com/podcast/.

[B45-jintelligence-08-00040] Kuncel Nathan R., Hezlett Sarah A., Ones Deniz S. (2004). Academic performance, career potential, creativity, and job performance: Can one construct predict them all?. Journal of Personality and Social Psychology.

[B46-jintelligence-08-00040] Lewis Neil, Wai Jonathan (2020). Communicating What we Know, and What Isn’t So: Science Communication in Psychology. Perspectives on Psychological Science.

[B47-jintelligence-08-00040] Lyall Catherine (2019). Being an Interdisciplinary Academic: How Institutions Shape Careers.

[B48-jintelligence-08-00040] Maranto Robert, Wai Jonathan (2020). Why intelligence is missing from American education policy and practice, and what can be done about it. Journal of Intelligence.

[B49-jintelligence-08-00040] Martschenko Daphne, Trejo Sam, Domingue Benjamin W. (2019). Genetics and education: Recent developments in the context of an ugly history and an uncertain future. AERA Open.

[B50-jintelligence-08-00040] Merton Robert K. (1973). The Sociology of Science: Theoretical and Empirical Investigations.

[B51-jintelligence-08-00040] National Academies of Sciences, Engineering and Medicine (2017). Communicating Science Effectively: A Research Agenda.

[B52-jintelligence-08-00040] Neisser Ulric, Boodoo Gwyneth, Bouchard Thomas J., Boykin A. Wade, Brody Nathan, Ceci Stephen J., Halpern Diane F., Loehlin John C., Perloff Robert, Sternberg Robert J. (1996). Intelligence: Knowns and unknowns. American Psychologist.

[B53-jintelligence-08-00040] Open Science Collaboration (2015). Estimating the reproducibility of psychological science. Science.

[B54-jintelligence-08-00040] Pinker Steven (2009). Malcolm Gladwell, Eclectic Detective. The New York Times.

[B55-jintelligence-08-00040] Resnick Brian (2016). Why IQ Matters More Than Grit. https://www.vox.com/2016/5/25/11683192/iq-testing-intelligence.

[B56-jintelligence-08-00040] Rindermann Heiner, Becker David, Coyle Thomas R. (2020). Survey of expert opinion on intelligence: Intelligence research, expert’s background, controversial issues, and the media. Intelligence.

[B57-jintelligence-08-00040] Ritchie Stuart (2016). Intelligence: All that Matters.

[B58-jintelligence-08-00040] Schmidt Frank L., Hunter John (2004). General mental ability in the world of work: Occupational attainment and job performance. Journal of Personality and Social Psychology.

[B59-jintelligence-08-00040] Sternberg Robert J. (2005). There are no public-policy implications: A reply to Rushton and Jensen. Psychology, Public Policy, and Law.

[B60-jintelligence-08-00040] Thompson Lee Anne, Oehlert Jeremy (2010). The etiology of giftedness. Learning and Individual Differences.

[B61-jintelligence-08-00040] Tseng Vivian (2012). The uses of research in policy and practice. Social Policy Report: Society for Research in Child Development.

[B62-jintelligence-08-00040] Tyack David B., Cuban Larry (1995). Tinkering toward Utopia.

[B63-jintelligence-08-00040] University of Chicago (1967). Kalven Committee: Report on the University’s Role in Political and Social Action. https://provost.uchicago.edu/sites/default/files/documents/reports/KalvenRprt_0.pdf.

[B64-jintelligence-08-00040] Wai Jonathan, Miller David I. (2015). Here’s Why Academics Should Write for the Public. The Conversation.

[B65-jintelligence-08-00040] Wai Jonathan, Worrell Frank C. (2015). Helping disadvantaged and spatially talented students fulfill their potential: Related and neglected national resources. Policy Insights from the Behavioral and Brain Sciences.

[B66-jintelligence-08-00040] Wai Jonathan, Brown Matt I., Chabris Christopher F. (2018). Using standardized test scores to include general cognitive ability in education research and policy. Journal of Intelligence.

[B67-jintelligence-08-00040] Wai Jonathan, Brown Matt, Chabris Christopher (2019). No One Likes the SAT. It’s Still the Fairest Thing about Admissions. The Washington Post.

[B68-jintelligence-08-00040] Warne Russell T. (2015). Five reasons to put the *g* back into giftedness: An argument for applying the Cattell-Horn-Carroll theory of intelligence to gifted education research and practice. Gifted Child Quarterly.

[B69-jintelligence-08-00040] Warne Russell T., Astle Mayson C., Hill Jessica C. (2018). What do undergraduates learn about human intelligence? An analysis of introductory psychology textbooks. Archives of Scientific Psychology.

[B70-jintelligence-08-00040] Whitehurst Grover J. (2019). A Prevalence of “Policy-Based Evidence Making.”. EducationNext.

[B71-jintelligence-08-00040] Woolston Chris (2020). Junior researchers hit by coronavirus-triggered hiring freezes. Nature.

[B72-jintelligence-08-00040] Yuhas Daisy (2020). A New Way of Predicting Which Kids Will Succeed in School: Look at Their Genes. NBC News.

[B73-jintelligence-08-00040] Zimmer Robert J., Akeel Bilgrami, Jonathan R. Cole (2015). What is academic freedom for?. Who’s Afraid of Academic Freedom?.

